# Understanding the Low Photosynthetic Rates of Sun and Shade Coffee Leaves: Bridging the Gap on the Relative Roles of Hydraulic, Diffusive and Biochemical Constraints to Photosynthesis

**DOI:** 10.1371/journal.pone.0095571

**Published:** 2014-04-17

**Authors:** Samuel C. V. Martins, Jeroni Galmés, Paulo C. Cavatte, Lucas F. Pereira, Marília C. Ventrella, Fábio M. DaMatta

**Affiliations:** 1 Departamento de Biologia Vegetal, Universidade Federal de Viçosa, Viçosa, MG, Brazil; 2 Research Group on Plant Biology under Mediterranean Conditions, Departament de Biologia, Universitat de les Illes Balears, Ctra. de Valldemossa, Palma, Balearic Islands, Spain; University of Antwerp, Belgium

## Abstract

It has long been held that the low photosynthetic rates (*A*) of coffee leaves are largely associated with diffusive constraints to photosynthesis. However, the relative limitations of the stomata and mesophyll to the overall diffusional constraints to photosynthesis, as well as the coordination of leaf hydraulics with photosynthetic limitations, remain to be fully elucidated in coffee. Whether the low actual *A* under ambient CO_2_ concentrations is associated with the kinetic properties of Rubisco and high (photo)respiration rates also remains elusive. Here, we provide a holistic analysis to understand the causes associated with low *A* by measuring a variety of key anatomical/hydraulic and photosynthetic traits in sun- and shade-grown coffee plants. We demonstrate that leaf hydraulic architecture imposes a major constraint on the maximisation of the photosynthetic gas exchange of coffee leaves. Regardless of the light treatments, *A* was mainly limited by stomatal factors followed by similar limitations associated with the mesophyll and biochemical constraints. No evidence of an inefficient Rubisco was found; rather, we propose that coffee Rubisco is well tuned for operating at low chloroplastic CO_2_ concentrations. Finally, we contend that large diffusive resistance should lead to large CO_2_ drawdown from the intercellular airspaces to the sites of carboxylation, thus favouring the occurrence of relatively high photorespiration rates, which ultimately leads to further limitations to *A*.

## Introduction

Because CO_2_ influx and water vapour efflux share a common pathway through the stomatal pores on leaf surfaces, a trade-off between transpirational costs and CO_2_ assimilation is implicitly unavoidable. The coupling between stomatal conductance (*g*_s_) to CO_2_ and water vapour (and the need to maintain a proper leaf water balance) has often been evidenced by the strong positive scaling between *g*_s_ and the leaf hydraulic conductance per unit area, *K*_L_ [1]–[3]. In turn, the significance of *K*_L_ as a potentially limiting component of the vascular system has been further emphasised by the strong hydraulic-photosynthetic coordination observed across a large sample of diverse species [4]. Furthermore, *K*_L_ is closely related to the anatomy of the leaf: *K*_L_ has been shown to be positively related to both the theoretical axial conductivity of the midrib (determined from xylem conduit numbers and dimensions) and the venation density, *D*_v_ [3], [5]. A unified control of hydraulic and photosynthetic traits may also be further highlighted by comparing shade and sun leaves: the former have lower rates of net CO_2_ assimilation (*A*) and *g*_s_, therefore leading to lower demand for water and correspondingly lower *K*_L_ and *D*_v_ [4], [6].

In addition to stomatal limitations, *A* is currently known to be constrained by mesophyll conductance (*g*_m_), which is defined as the conductance for the transfer of CO_2_ from the intercellular airspaces (*C*_i_) to the sites of carboxylation in the chloroplastic stroma (*C*_c_) [7]. According to Flexas et al. [8], *g*_m_ limitations to photosynthesis are of similar magnitude as stomatal constraints and generally greater than biochemical limitations. Increasing evidence has shown that *g*_m_ is often intrinsically co-regulated with *g*_s_ [8]. More recently, Ferrio et al. [9] showed a positive scaling between *g*_m_ and *K*_L_ and proposed that water and CO_2_ share an important portion of their respective diffusion pathways through the mesophyll; thus, any downregulation of leaf hydraulics may reduce not only *g*_s_ but also *g*_m_, both of which contribute to reducing *A*. Quantification of *g*_m_ has become important in predicting leaf photosynthetic parameters using the Farquhar-von Caemmerer-Berry (FvCB) model of leaf photosynthesis [10] because such a model underestimates the maximum Rubisco carboxylation rate (*V*_cmax_) by considering *g*_m_ as being infinite [11], [12]. Furthermore, changes in *C*_i_ –*C*_c_ because of variations in *g*_m_ among species result in different *A* values in plants with the same biochemical activity and *g*_s_ [7].

At lower *C*_c_, *A* is limited by RuBP carboxylase activity, which in turn depends on the concentration, activation and kinetic properties of Rubisco [13]. For example, Rubisco with a higher specificity factor, *S*_c/o_ (which determines the relative rates of carboxylation and oxygenation by Rubisco at given CO_2_ and O_2_ concentrations), could be regarded as conferring an advantage in minimising photorespiration rates, *R*_P_ [14]. Interestingly, Rubisco seems to have evolved towards higher *S*_c/o_ under stressful conditions leading to low *C*_c_, such as drought, salinity and high temperature [14]. Additionally, species evolved under stressful conditions with sclerophyll leaves tend to display a higher *S*_c/o_, which may again be related to lower *C*_c_ [14], [15]. However, trade-offs between *S*_c/o_ (or particularly the affinity for CO_2_ (1/*K*_C_)) and the carboxylase turnover rate (*k*_cat_^c^) have been described, so that there is no Rubisco in nature with both high affinity for CO_2_ and fast activity [16]. At saturating *C*_c_, *A* becomes limited by the capacity for the regeneration of RuBP (often dominated by the electron transport capacity) but also by *S*_c/o_ [17]. In any case, when studying the ecophysiology of plants, Rubisco kinetic parameters should not be considered a constant but rather as an active part determining the biochemical potential of plants to assimilate CO_2_ under optimal and suboptimal conditions.

Coffee is an evergreen perennial shrub with hypostomatous leaves that evolved in the African forest understoreys and is thus considered a shade-demanding species. However, in many situations, modern coffee cultivars grow well without shade and even out-yield shaded coffee [18], [19]. At atmospheric CO_2_ concentrations and saturating light, coffee displays low *A*, typically in the range of 4–11 µmol CO_2_ m^−2^ s^−1^ [20], [21], which is in the lower range recorded for trees [22]. However, the maximum *A* values obtained in common *A/C*_i_ curves can surpass 20 µmol CO_2_ m^−2^ s^−1^ [23], whereas the photosynthetic capacity, determined under true CO_2_ saturation (∼50 mmol CO_2_ mol^−1^ air), reaches values exceeding 30 µmol O_2_ m^−2^ s^−1^ [21]. Taking the above information together, the low *A* in coffee might largely result from diffusive constraints to photosynthesis [24]. However, the relative contributions of the stomata and mesophyll to the overall diffusional limitations to photosynthesis, as well as the coordination of leaf hydraulics with photosynthetic limitations, remain to be fully elucidated in coffee. Furthermore, the anticipated low *g*_s_ and *g*_m_ would compromise the transference of CO_2_ through stomata and leaf mesophyll to Rubisco sites, therefore decreasing *C*_c_ and ultimately favouring RuBP oxygenation in relation to carboxylation [25]. Under these conditions, Rubisco kinetic properties with a high affinity for CO_2_ may be critical to secure optimal carbon balance and yield.

Although the general descriptors of the ecophysiology of sun-shade plants are well-known, an integrative analysis of these descriptors has rarely been performed in conjunction (hydraulics, stomata, mesophyll, Rubisco, etc). Here, we provide a holistic analysis to understand the causes associated with low *A* in coffee by measuring a variety of key anatomical/hydraulic and photosynthetic traits, including *D*_v_, *K*_L_, the actual and maximum theoretical *g*_s_ (*g*_wmax_) and several parameters from *A*/*C*_i_ and *A*/*C*_c_ curves. We have centred our attention on coffee given that, in addition to being a highly important commodity [18], it could also be considered as a model plant for other important crops which evolved as understorey trees, such as cacao, citrus and tea; these crops are traditionally considered to have low *A* (seldom above 10 µmol CO_2_ m^−2^ s^−1^, even in the field under favourable conditions) likely as consequence of large diffusive, rather than biochemical, limitations to photosynthesis [26]. Our main goals were (i) to examine the role of leaf hydraulics as a prime limiting factor for photosynthetic gas exchange, (ii) to calculate *g*_m_ to properly parameterise the responses of *A* to *C*_c_, and (iii) to disentangle the relative contributions of stomatal, mesophyll and biochemical limitations to photosynthesis, and how all of these facts may be affected by the light supply. A further goal was to analyse how the kinetic properties of coffee’s Rubisco [27] may affect the actual *A* and evaluate if Rubisco in coffee is well tuned for operating at low *C*_c_ by comparing it with the highly efficient Rubisco of *Limonium gibertii*; this shade-intolerant species is particularly adapted to highly stressing environments, whose Rubisco has evolved under extremely low *C*_c_ ranges [14]. We demonstrate that leaf hydraulic architecture imposes a major constraint on the maximisation of the photosynthetic gas exchange of coffee leaves and that *A* was mainly limited by stomatal factors followed by similar limitations associated with mesophyll and biochemical constraints, regardless of the light supply. We also found that, despite the relatively high *S*_c/o_ of coffee Rubisco, the large diffusive resistances favour the occurrence of relatively high *R*_P_, which ultimately leads to further limitations to *A*.

## Materials and Methods

### Plant Material, Growth Conditions and Experimental Design

The experiment was conducted in Viçosa (20°45′S, 42°54′W, altitude 650 m) in southeastern Brazil. Uniform seedlings of *Coffea arabica* L. cv ‘Catuaí Vermelho IAC 44′ obtained from seeds were grown in 12 L pots containing a mixture of soil, sand and composted manure (4∶1∶1, v/v/v). Plants were grown either under full sunlight conditions (100% light) or under low light in a shade environment (10% full sunlight). The shade enclosure was constructed using neutral-density black nylon netting, and the plants were kept in these conditions for 12 months before measurements. Throughout the experiment, the plants were grown under naturally fluctuating conditions of temperature and relative air humidity and were fertilised and irrigated as necessary. The pots were randomised periodically to minimise any variation within each light environment. For all samplings and measurements, the youngest fully expanded leaves, corresponding to the third or fourth pair from the apex of the plagiotropic branches, were used. The experiment was arranged in a completely randomised design, with six plants in individual pots per treatment as replicates. The experimental plot included one plant per container.

### Morpho-anatomical Features and Related Hydraulic and Diffusive Traits

The specific leaf area (SLA) was computed using the dry mass of 20 leaf discs (1.13 cm diameter each). For anatomical measurements, leaves were collected and fixed in FAA_70_ for 48 h, followed by storage in 70% (v/v) aqueous ethanol. Samples of the mean region of each leaf blade were embedded in methacrylate (Historesin–Leica Microsystems Nussloch, Heidelberg, Germany) according to the manufacturer. Transverse sections (7 µm thickness), obtained using a rotary microtome (model RM2155, Leica Microsystems Inc., Deerfield, USA), were stained with toluidine blue at pH 4.0 and mounted in synthetic resin (Permount). Anatomical data were quantified using an image analysis program (Image Pro-Plus, version 4.5, Media Cybernetics, Silver Spring, USA). Video images were acquired using a video camera attached to an Olympus microscope (AX70TRF, Olympus Optical, Tokyo, Japan). The following anatomical data were then assessed: (i) total leaf thickness; (ii) palisade and spongy mesophyll thickness; (iii) upper and lower epidermis thickness; (iv) guard cell length (*L*); (v) vertical distance from the vein to stomatal epidermis, *D*_v-e_; (vi) stomatal density (SD, according to DaMatta et al. [28]); (vii) stomatal index that was estimated as 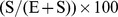, where S and E are the number of stomata and epidermal cells per unit leaf area, respectively [29]; and (viii) stomatal pore index based on guard cell length (SPI_gcl_) that was estimated as 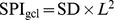. For determining venation traits, samples of leaf lamina were cut from two leaves per plant and cleared as described by Scoffoni et al. [30]. Regions of approximately 6 mm^2^ were imaged at 30X, and *D*_v_ was calculated as the sum of the vein lengths divided by the total image area using the image analysis program described above. Leaf size was estimated, using the maximum leaf widths and lengths, with the equations described by Antunes et al. [31]. Stomatal size and epidermal cell size, as well as the level of coordination between anatomical traits and leaf size (quantified as the deviation from the expected proportional relationship to each other), were all determined following Carins Murphy et al. [6].

Midrib xylem conduits were measured to determine the theoretical midrib axial hydraulic conductance (*K*_t_), where the conduits were treated as ellipses to calculate *K*_t_ as
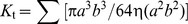
where *a* and *b* are the long and short internal vessel diameters, and η is the viscosity of water at 25°C [32], further normalising by leaf length and area.

The maximum stomatal conductance to water vapour (*g*_wmax_) was calculated according to Franks et al. [33] as
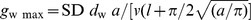
where SD is the stomatal density, *d*_W_ is the diffusivity of water vapour in air, *a* is the maximum area of the open stomatal pore, *v* is the molar volume of air, and *l* is the stomatal pore depth for fully open stomata. Values for standard constants *d*_W_ and *v* were those for 25°C (24.9×10^−6^ m^2^ s^−1^ and 24.4×10^−3^ m^3^ mol^−1^, respectively). *a* was calculated as 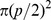, where *p* is the stomatal pore length, which was approximated as *L*/2 according to Franks and Farquhar [34]. *l* for fully open stomata was taken as *L*/4, assuming guard cells inflate to a circular cross section [33].

### Leaf Hydraulic Conductance

The leaf hydraulic conductance (*K*_L_) was estimated according to Brodribb and Holbrook [35] by following the kinetics of water potential (Ψ_l_) relaxation in rehydrated leave as:
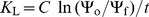
where *C* is leaf capacitance, estimated using pressure-volume curves [36], Ψ_o_ is Ψ_l_ before rehydration, and Ψ_f_ is Ψ_l_ after rehydration for *t* seconds. Leaf Ψ_l_ was measured using a Scholander-type pressure chamber (model 1000, PMS Instruments, Albany, NY, USA).

### Gas Exchange and Fluorescence Measurements

Leaf gas exchange and chlorophyll *a* fluorescence were measured simultaneously using an open-flow infrared gas-exchange analyser system equipped with a leaf chamber fluorometer (LI-6400XT, Li-Cor, Lincoln, NE, USA). Environmental conditions in the leaf chamber consisted of a leaf-to-air vapour pressure deficit between 1.2 and 2.0 kPa and a leaf temperature of 25°C.

In light-adapted leaves, the actual quantum yield of PSII (Φ_PSII_) was determined by measuring steady-state fluorescence (*F*_s_) and maximum fluorescence during a light-saturating pulse of *c*. 8,000 µmol m^−2^ s^−1^ (*F*_m_′), following the procedures of Genty et al. [37]:
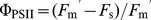



The electron transport rate (*J*_F_) was then calculated as
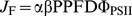
where PPFD is the photosynthetically active photon flux density, α is the leaf absorptance and β is the PSII optical cross section. The product α β was herein determined from the relationship between Φ_PSII_ and Φ_CO2_, obtained by varying the CO_2_ concentration under non-photorespiratory conditions in an atmosphere containing less than 1% O_2_, as described by Valentini et al. [38].

The light respiration rate (*R*_L_) was determined according to the ‘Laisk-method’ [39], using the *y* axis intersection of *A*/*C*_i_ (internal CO_2_ concentration) curves performed at three different PPFD intensities (750, 250 and 75 µmol m^−2^ s^−1^). The rate of mitochondrial respiration at darkness (*R*_D_) was measured early in the morning in dark-adapted leaves.

The photorespiratory rate of Rubisco (*R*_P_) was obtained according to Valentini et al. [38] using the following equation:
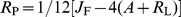



Six *A*/*C*_i_ curves were obtained from different plants per treatment. In light-adapted leaves, *A*/*C*_i_ curves were initiated at an ambient CO_2_ concentration (*C*_a_) of 400 µmol mol^–1^ under a saturating PPFD of 1000 µmol m^–2^ s^–1^. Once steady state was reached, *C*_a_ was decreased stepwise to 50 µmol mol^–1^ air. Upon completion of the measurements at low *C*_a_, *C*_a_ was returned to 400 µmol mol^–1^ air to restore the original *A*. Next, *C*_a_ was increased stepwise to 2,000 µmol mol^–1^ air. *A*/*C*_i_ curves consisted of 13 different *C*_a_ values.

*C*_c_ was calculated after Harley et al. [40] as
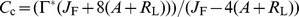
where Г^*^ was determined from the *in vitro* Rubisco specificity factor (*S*_c/o_) as



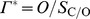



*A* was taken from gas-exchange measurements, and the *J*_F_ values were obtained from chlorophyll *a* fluorescence yield. After estimating *C*_c_, *g*_m_ was calculated following Harley et al. [40]:
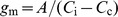



From the *A*/*C*_i_ and *A*/*C*_c_ curves, the maximum carboxylation capacity (*V*_cmax_) and maximum capacity for electron transport rate (*J*_max_) were calculated on a *C*_i_ and *C*_c_ basis using the kinetic parameters for coffee described in Martins et al. [27]. The FvCB model was fitted to the data by applying iterative curve fitting (minimum least square difference) using the Microsoft Excel Solver tool (Microsoft Corporation, Redmond, WA, USA). Additionally, *g*_m_, *V*_cmax_ and *J*_max_ were estimated using the Ethier and Livingston [11] method, which is based on fitting *A*/*C*_i_ curves with a non-rectangular hyperbola version of the FvCB model, relying on the hypothesis that *g*_m_ reduces the curvature of the Rubisco-limited portion of an *A*/*C*_i_ response curve. Again, the kinetic parameters of Rubisco measured on coffee were used. Corrections for the leakage of CO_2_ and water vapour into and out of the leaf chamber of the Li-6400-40 have been applied to all gas-exchange data, as described by Rodeghiero et al. [41].

The chloroplastic CO_2_ concentration of transition (*C*_c_trans_), where *C*_c_ denotes the transition between the Rubisco- and RuBP regeneration-limited states, was estimated as described by Gu et al. [42]:
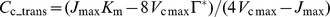
where *K*_m_ is the effective Michaelis–Menten constant for CO_2_ that considers the competitive inhibition by O_2_ which was taken from Martins et al. [27].

The overall photosynthetic limitations were partitioned into their functional components [stomatal (*l*_s_), mesophyll (*l*_m_) and biochemical (*l*_b_) limitations] using the values of *g*_s_, *g*_m_, *V*_cmax_, Г^*^, *K*_m_ and *C*_c_ following the approach proposed by Grassi and Magnani [43]:
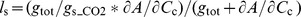


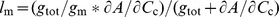


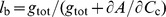
where *g*_s_CO2_ is the stomatal conductance to CO_2_ (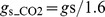), *g*_m_ is the mesophyll diffusion conductance according to Harley *et al*. [40] and *g*_tot_ is the total conductance to CO_2_ from ambient air to chloroplasts (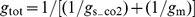). ∂*A*/∂*C*_c_ was calculated as:



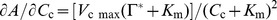



### Statistical Analyses

Data are expressed as the means ± standard error. Student’s *t*-tests were used to compare the parameters between treatments. Additionally, one sample *t*-tests were performed to compare the means for shade plants with the expected values if they were proportional to the sun plants. All statistical analyses were carried out using Microsoft Excel.

## Results

**Figure 1:**
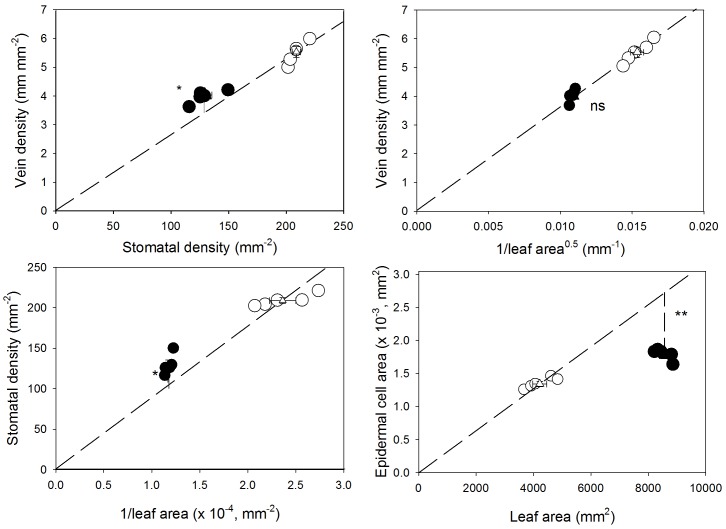
The relationships between vein and stomatal density, vein density and 1/√ leaf area, stomatal density and 1/leaf area, and epidermal cell area and leaf area. Data (± standard error) are shown for coffee plants grown under shade (black circles) or full sun (white circles). Asterisks denote differences (*, *P*<0.05; **, *P*<0.01) between the observed values for the shade plants and the expected values (broken line) if these were proportional to the averages for the sun plants.

Sun- and shade-grown individuals differed reasonably in venation architecture and mesophyll structure (Table 1). The sun leaves, compared with shade leaves, displayed a higher leaf thickness (15%) primarily resulting from a higher thickness of palisade (43%) and spongy (14%) mesophyll, which led to a higher palisade-to-spongy parenchyma ratio (25%) and lower SLA (39%). However, the upper and lower epidermis thickness and the maximum distance from the vein to the epidermis (*D*_v-e_) did not change in response to the light treatments. Sun-grown plants had 31% more midrib xylem conduits but with slightly lower mean conduit diameters (7%) than shade-grown plants. The sun plants displayed *K*_t_ and *K*_L_ values that were 160% and 58% higher, respectively, than in the shade individuals. Adjustments to the light treatments associated with the morphological characteristics of stomata were also observed, as denoted by higher SD (62%), stomatal index (27%) and SPI_gcl_ (62%) in sun leaves compared with the shade leaves; however, both the guard cell length and stomatal size did not change in response to the light treatments. Vein density was proportional to 1/√ leaf area but slightly higher (17%) than would be expected if it was directly proportional to SD (Figure 1). In line with the differences in the stomatal index, SD and epidermal cell size deviated significantly from the expected proportional relationship to leaf area: SD was 25% higher and epidermal cell size was 31% lower in shade plants than would be expected if these variables were directly proportional (Figure 1). The maximum *g*_s_ (*g*_wmax_), determined by the stomatal size and density, was larger (61%) in sun leaves than in the shade leaves.

**Table 1:** Anatomical and hydraulic traits of coffee plants grown under shade or full sunlight conditions.

Parameters	Treatments	
	Shade	Full sunlight
Specific leaf area (m^2^ kg^−1^)	22.9±3.5	14.0±2.7*
Total leaf thickness (µm)	333.9±9.1	384.5±17.2*
Palisade thickness (µm)	52.6±1.2	75.24±4.4*
Spongy thickness (µm)	220.9±6.1	253.1±11.5*
Upper epidermis thickness (µm)	38.8±0.2	37.4±1.3
Lower epidermis thickness (µm)	29.6±0.9	26.4±1.2
PP/SP	0.24±0.01	0.30±0.01*
SPI_gcl_	0.104±0.006	0.168±0.003*
Guard cell length (µm)	28.0±0.7	28.9±0.4
Stomatal density (mm^2^)	129.1±7.2	208.8±4.2*
Stomatal index	20.3±1.1	25.7±1.0*
*g*_wmax_ (mol m^−2^ s^−1^)	1.16±0.05	1.82±0.02*
Venation density (mm mm^−2^)	4.0±0.1	5.5±0.2*
Midrib vessel diameter (µm)	22.8±0.2	21.3±0.4*
Number of midrib conduits	117±7	153±11*
*K*_t_ (mmol MPa^−1^ m^−2^ s^−1^)	24.5±2.5	63.8±1.6*
*K*_L_ (mmol MPa^−1^ m^−2^ s^−1^)	6.9±0.1	10.9±1.4*
*D*_v-e_ (µm)	195.8±8.6	215.4±8.5

*n = *6±SE. Asterisks indicate statistically significant differences (*P*<0.05) between shade and full sun treatments.

Abbreviations: PP:SP, palisade-to-spongy parenchyma ratio; SPI_gcl_, stomatal pore index based on guard cell length; *g*_wmax_, maximal theoretical stomatal conductance to water vapour; *K*_t_, midrib conductance; *K*_L_, leaf hydraulic conductance; *D*_v-e_, vertical distance from the vein to the stomatal epidermis.

Under saturating PPFD (1,000 µmol photons m^−2^ s^−1^), both *A* and *g*_s_ values were higher (*c.* 55%) in sun leaves than in the shade leaves (Table 2). The values for several other photosynthetic/respiration parameters were also higher in sun leaves than in the shade leaves (Table 2): 60% for *g*_m_ (average of two independent methods), 42% for *V*_cmax_, 45% for *J*_max_ (for *V*_cmax_ and *J*_max_, the values express averages obtained on *C*_i_ and *C*_c_ bases), 140% for *R*_D_, 200% for *R*_L_, 41% for *R*_p_ and 51% for *J*_F_. In contrast, *C*_i_, *C*_c_, *C*_c_trans_ and the ratios *A*/*g*_s_, *g*_m_/*g*_s_ and *J*_max_/*V*_cmax_ on both *C*_i_ and *C*_c_ bases did not differ significantly in response to the light treatments. *V*_cmax_ and *J*_max_ calculated on a *C*_c_ basis were, on average, 101% and 37% higher than *V*_cmax_ and *J*_max_, respectively, calculated on a *C*_i_ basis, whereas the *J*_max_/*V*_cmax_ ratio was on average 32% lower when calculated on a *C*_c_ basis than on a *C*_i_ basis.

**Table 2:** Mean values for the photosynthetic and respiration parameters of coffee plants grown under shade or full sunlight conditions.

Parameters	Treatments	
	Shade	Full Sun
*A* (µmol CO_2_ m^–2^ s^–1^)	7.9±0.4	12.0±0.8*
g_s_ (mmol H_2_O m^–2^ s^–1^)	94±9	146±17*
*g*_m_Harley_ (mmol CO_2_ m^–2^ s^–1^)	76±4	116±10*
*g*_m_Ethier_ (mmol CO_2_ m^–2^ s^–1^)	65±12	109±5*
*C*_i_ (µmol CO_2_ mol^–1^ air)	247±11	246±7
*C*_c_Harley_ (µmol CO_2_ mol^–1^ air)	143±10	142±9
*J*_F_ (µmol e^−^ m^–2^ s^–1^)	70±5	106±5*
*V*_cmax_*C*i_ (µmol CO_2_ m^–2^ s^–1^)	26.8±1.6	42.1±2.2*
*V*_cmax_*C*c_ (µmol CO_2_ m^–2^ s^–1^)	58.1±3.5	78.5±4.0*
*J*_max_Ci_ (µmol e^–^ CO_2_ m^–2^ s^–1^)	71.1±4.4	109.5±3.1*
*J*_max_Cc_ (µmol e^–^ m^–2^ s^–1^)	102.7±6.3	142.6±7.4*
*J*_max_/*V*_cmax_*C*i_	2.6±0.04	2.7±0.1
*J*_max_/*V*_cmax_*C*c_	1.8±0.1	1.8±0.1
*C*_c_trans_ (µmol CO_2_ mol^–1^ air)	228±17	249±23
*R*_D_ (µmol CO_2_ m^–2^ s^–1^)	0.5±0.06	1.2±0.05*
*R*_L_ (µmol CO_2_ m^–2^ s^–1^	0.1±0.05	0.3±0.08*
*A*/*g*_s_ (µmol CO_2_ mol^–1^ H_2_O)	86±6	84±4
*g*_m_Harley_/*g*_s_ (mol CO_2_ mol^–1^ CO_2_ )	1.4±0.1	1.3±0.1
*R*_p_ (µmol CO_2_ m^–2^ s^–1^)	3.2±0.4	4.5±0.1*
Stomatal limitation	0.41±0.04	0.38±0.02
Mesophyll limitation	0.30±0.02	0.30±0.01
Biochemical limitation	0.29±0.03	0.32±0.03

*n = *6±SE. The overall photosynthetic limitations associated with stomatal, mesophyll and biochemical factors are also shown. Data for *A*, *g*_s_, *g*_m_Harley_, *C*_i_, *C*_c_Harley_, *J*_F_, and *R*_P_ were obtained under PPFD of 1000 µmol m^−2^ s^−1^, *C*_a_ of 400 µmol mol^−1^ and leaf temperature of 25°C. Asterisks indicate statistically significant differences (*P*<0.05) between shade and full sun treatments.

Abbreviations: *A*, net photosynthesis; *g*_s_, stomatal conductance to water vapour; *g*_m_, mesophyll conductance to CO_2_; *C*_i_, sub-stomatal CO_2_ concentration; *C*_c_, chloroplastic CO_2_ concentration; *J*_F_, electron transport rate estimated by chlorophyll fluorescence; *V*_cmax_, maximum carboxylation capacity; *J*_max_, maximum capacity for electron transport rate; *C*_c_trans_, the *C*_c_ that denotes the transition between the Rubisco- and RuBP regeneration-limited state; *R*_D,_ dark respiration; *R*_L_, light respiration; *R*_P,_ photorespiration rate.

The overall photosynthetic limitations were essentially similar when comparing sun- and shade-grown plants (Table 2). We found that *A* was mainly constrained by stomatal limitations (*c.* 40%), whereas mesophyll and biochemical limitations accounted similarly for the remaining limitations (*c.* 30% each).

The analysis of Rubisco kinetic properties are summarised in Table 3, showing that coffee presents a Rubisco with a relatively high affinity for CO_2_ (low *K*_c_) and fast activity (*k*_cat_^c^), resulting in relatively high values for *S*_c/o_. From the combination of *in vivo V*_cmax_ and *in vitro* data (*k*_cat_^c^), the concentration of active Rubisco sites was estimated to be 16 and 20 µmol m^−2^ s^−1^ for shade and sun plants, respectively.

**Table 3:** Rubisco kinetic constants measured for coffee (taken from Martins et al. [27]).

Species	*S*_c/o_	Γ* (µbar)	*K*_c_ (µM)	*K*_o_ (µM)	*k*_cat_^c^(s^−1^)
Coffee	98.4±4.3	39.6±1.7	10.3±1.3	479±113	3.2±0.1

*n* = 4±SE.

Abbreviations: *S*_c/o_, Rubisco specificity factor; Γ*, CO_2_ compensation point in the absence of mitochondrial respiration; *K*_c_ and *K*_o_, the Michaelis-Menten kinetics for CO_2_ and O_2_, respectively; *k*_cat_^c^, Rubisco catalytic turnover rate for the carboxylase reaction.

## Discussion

This study provided a holistic examination of the key steps that could limit the photosynthetic capacity to fix CO_2_ and demonstrated that leaf hydraulic architecture imposes a major constraint on the maximisation of the photosynthetic gas exchange of coffee leaves by limiting *g*_s_. It is tempting to suggest that these constraints might to some extent explain the photosynthetic behaviour of other important (sub)tropical crops such as cacao, citrus and tea, which have a photosynthetic performance and a slow-growth behaviour similar to that of coffee [26]. Our results suggest that improvements of the photosynthetic performance of these crops by means of selecting hydraulic traits (e.g., *D*_v_ and *K*_L_) that might support higher *g*_s_ values (thereby decreasing stomatal limitations of photosynthesis) could be a useful strategy to facilitate the selection of promising genotypes with enhanced crop growth and production.

We have shown that adjustments in leaf hydraulics through increases in *D*_v_ and *K*_L_ in sun-grown individuals were coordinated with a suite of traits related to water flux and gas exchange per leaf area, such as mesophyll structure (e.g., higher mesophyll thickness and palisade-to-spongy parenchyma ratio) and stomatal attributes (increased SD and stomatal pore index). Importantly, we showed that increases in *K*_t_ occurred at the expense of an increased number of midrib conduits with lower lumen in sun plants, suggesting that improvements in the hydraulic safety would not compromise the hydraulic efficiency [44]. In addition, the larger SD coupled with larger stomatal index implies a proportionally greater increase in the number of guard cells than in normal epidermal cells [45]; hence, light availability should directly and positively influence stomatal fate in coffee regardless of passive changes linked by light-induced differences in leaf blade expansion. However, differential leaf expansion was responsible for the adjustment of *D*_v_ to SD, which remained nearly proportional to each other. Thus, coffee plants can balance water supply with transpirational demand through a coordination of increased initiation of stomata cells with differential expansion of epidermal cells. Such coordination reflects an optimisation of the trade-off between transpirational costs and CO_2_ assimilation, resulting in the higher intrinsic water use efficiency observed in coffee (*c.* 85 µmol CO_2_ mol^−1^ H_2_O on average), which is markedly high compared with other tropical woody species [46], [47].

Our *K*_L_ values, determined using the method of relaxation kinetics of Ψ_l_, were remarkably lower than those found by Brodribb et al. [1] for other tropical trees using the same method (between 17 and 36 mmol m^−2^ s^−1^ MPa^−1^). Considerably low values of *K*_L_ had already been reported for *C. arabica* (*c.* 4.2 mmol m^−2^ s^−1^ MPa^−1^) by Gascó et al. [48] using the high-pressure method. Consistent with the close relationship between *K*_L_ and *D*_v_ [4], [5], [49], our maximum *D*_v_ values were also within the low range recorded for angiosperms [50], suggesting that the hydraulic architecture of coffee leaves imposes strong resistance for water flow which, in turn, should limit the CO_2_ diffusion into the leaf [4]. In addition, our *K*_L_ and *D*_v_ values (Table 1) fit very well in the general relationship of both *K*_L_ and *D*_v_ with the maximum *A* proposed by Brodribb et al. [5], [51], that is, our estimated *K*_L_ and *D*_v_ values exactly predict our maximum measured *A* values. Therefore we contend that the leaf hydraulic architecture ultimately should act as a prime factor limiting photosynthetic gas exchange in coffee leaves.

Regardless of light regimens, our actual *g*_s_ values were relatively similar to the value of 108 mmol H_2_O m^−2^ s^−1^ averaged over a range of studies using coffee plants grown under optimal conditions ([10], and references therein). These values are significantly lower than those of the modelled *g*_wmax_, resulting in a *g*_wmax_/*g*_s_ ratio above 13, as can be calculated for sun-grown plants. By comparison, that ratio was *c.* 2.5 in non-water-stressed tomato [52] and 4.5 in *Eucalyptus globulus* [33]. The high hydraulic resistance of the coffee leaves is most likely the cause of the difference between the theoretical *g*_wmax_ and the recorded *g*_s_. Nevertheless, this large difference raises the following questions: why would the plant invest in a large *g*_wmax_ if the maximum realisable *A* is relatively low and constrained by the leaf hydraulic architecture? Furthermore, from an ecological point of view, what would be the advantage of having a large *g*_wmax_ if, in the humid, shaded understoreys where coffee evolved, *A* should be more constrained by light limitation than by CO_2_ supply? Despite not having immediate responses to these queries, our results suggest a lack of coordination between the maximum capacity for stomatal aperture and carbon fixation, as also noted in saplings of Bornean rainforest tree species grown in the understorey [53]. A large *g*_wmax_ may not be problematic in terms of water loss in the humid understorey, where transpiration rates are expected to be much more dependent on boundary layer resistance, and thus the importance of the stomata in optimising photosynthetic gas exchange should be reduced. In any case, considering that bigger stomata tend to close slower than smaller stomata [33], [54], the relatively large stomata of coffee leaves (combined with low *K*_L_) might result in excessive leaf desiccation if large stomatal apertures are realised. In this sense, the low actual *g*_s_ might be a conservative strategy to maintain leaf hydration and minimise the risk of xylem embolism. This observation is in line with recent results obtained for *Toona ciliata*, where transpirational homeostasis to changes in vapour pressure deficit was achieved through dynamic stomatal control rather than modification of the relationship between veins and stomata [55]. Taken together, these findings lead to the interesting question of why long-term adjustments to the parameters that define *g*_smax_ have not been fixed and regulation of *g*_s_ comes predominantly from short-term adjustments to environmental conditions but at the cost of inherently low *g*_s_ in some species, such as coffee.

Our maximum *g*_m_ value was *c.* two-fold higher than those previously reported for *C. arabica* seedlings [56]. In any case, our *g*_m_ values were similar to those obtained for other evergreen woody species (e.g. [57]–[59]). Greater *g*_m_ values for sun leaves, as found here, have been systematically reported and have been often explained by anatomical and morphological differences between shade and sun leaves [60]. Despite the changes in *g*_m_ and *A* between shade- and sun-grown coffee plants, *C*_i_ and *C*_c_ remained fairly similar. Thus, regardless of the light treatments, when *A* changed, *g*_m_ and *g*_s_ scaled accordingly, and, hence, *C*_c_ remained constant [61]. This proportional scaling lends support to explain why stomatal, mesophyll and biochemical limitations to photosynthesis were similar between sun and shade leaves. These findings are in agreement with other studies, which show that the approximate scaling of *g*_s_ and *g*_m_ with *A* makes the relative limitations to photosynthesis rather conservative between sun and shade leaves, as also noted in *Fagus sylvatica* [62].

Irrespective of the light environment, the mean drawdown from *C*_i_ to *C*_c_ (*c.* 79 µmol mol^−1^) was lower than that from *C*_a_ to *C*_i_ (*c.* 153 µmol mol^−1^), which is consistent with the fact that the diffusive limitations to CO_2_ in mesophyll were lower than those in stomata, thus ultimately reflecting a high *g*_m_/*g*_s_ ratio. Indeed, by analysing the dataset reported by Flexas et al. [63], we noted that species operating at low *C*_i_ tended clearly to display an increased *g*_m_/*g*_s_ ratio (Figure S1) which contributes to an improved performance in photosynthetic water use [63]. We believe that such feature is essential for keeping a positive carbon balance given that stomatal limitations may even be exacerbated in coffee trees grown under field conditions, particularly because *g*_s_ peaks in the early morning and decreases progressively throughout the day, reaching values typically ranging from 10 to 50 mmol H_2_O m^−2^ s^−1^ from midday onwards [23], [24], [64]–[67] as a consequence of rising vapour pressure deficit.

Under saturating PPFD, *A* at ambient CO_2_ was limited by Rubisco activity regardless of the light treatment given that the estimated *C*_c_ was lower than *C*_c_trans_ (Table 2). However, the realised *A* and *J*_F_ at maximum growth irradiance of shade plants (*c*. 200 µmol PPFD m^−2^ s^−1^) are *c.* 60% of their saturating values (Figure S2), indicating that, even though these plants operate during most of their development under light limitation, the balance between RuBP regeneration and Rubisco activity (the *J*_max_/*V*_cmax_ ratio) was essentially similar in the shade and sun plants (Table 2). These data indicate that, in contrast to the optimal distribution principle, which suggests that Rubisco and the regeneration of RuBP should co-limit photosynthesis such that no excess capacities remain [68], the coffee plant does not properly optimise its resource allocation. On the one hand, under light saturation, an excess of electron transport capacity is to be expected, given that the actual *C*_c_ operates far from *C*_c_trans_ (*cf*. Table 2). On the other hand, under light limitation, a great investment in capacity for carbon assimilation and electron transport is retained despite the low realised *A* and *J*_F_ by the shade leaves.

**Figure 2:**
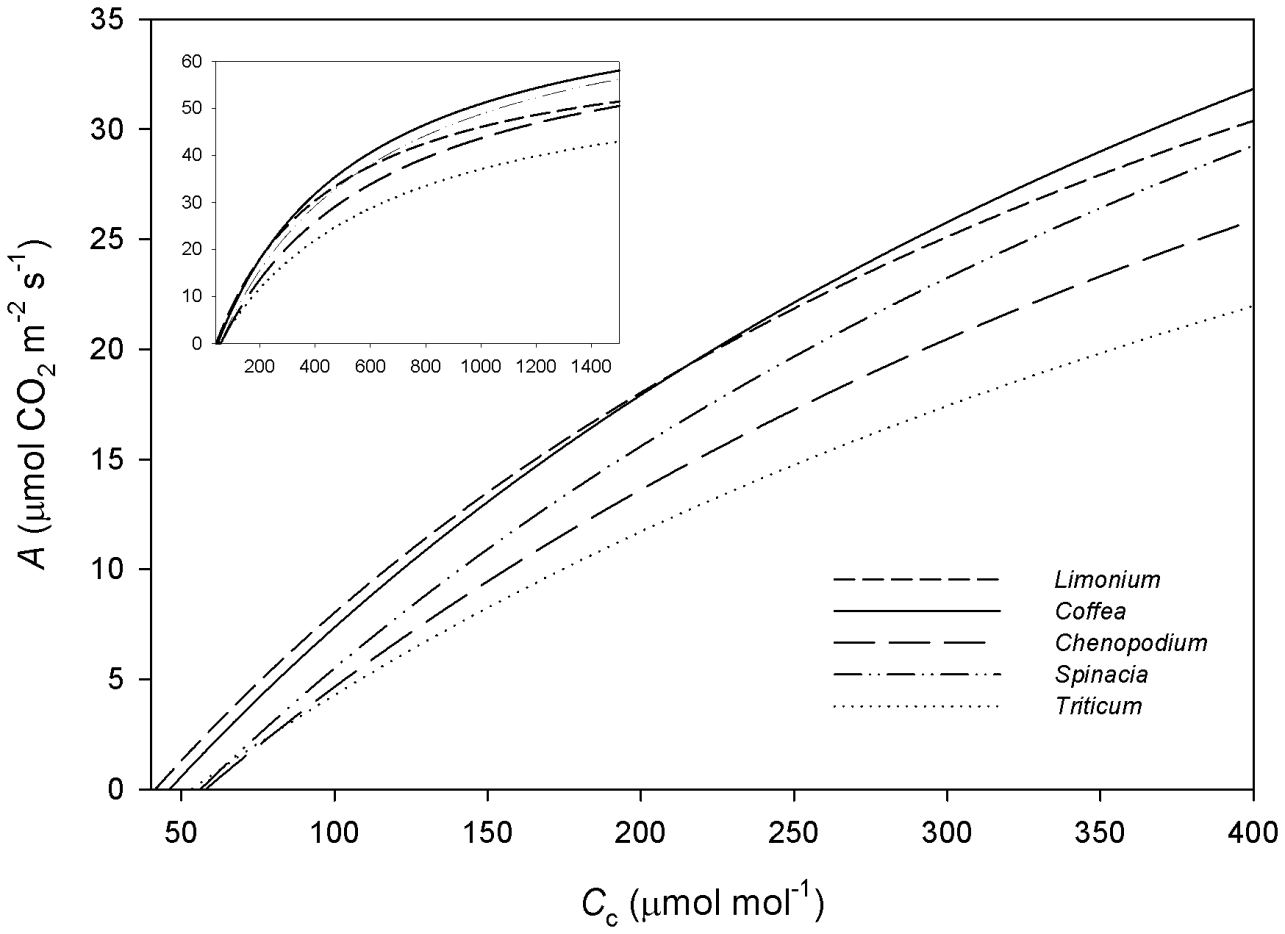
Comparison of the CO_2_ assimilation rate as a function of *C*_c_, modelled with the kinetic parameters of Rubisco in coffee and several C_3_ species. The RuBP saturated rates of CO_2_ assimilation at 25°C were calculated using the following equation [13]: 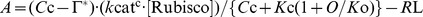. *C*_c_, the chloroplastic CO_2_ partial pressure; *O*, the intercellular O_2_ partial pressure; Γ*, the CO_2_ compensation point in the absence of day respiration; [Rubisco], the catalytic site content of Rubisco. The Rubisco kinetic properties for coffee (Table 3) and the kinetic properties for the other species were retrieved from Savir et al. [69] and are summarized in Table S1. The *R*_L_ and [Rubisco] were set as 1.0 µmol CO_2_ m^−2^ s^−1^ and 25 µmol sites m^−2^, respectively.

Coffee Rubisco was characterised as displaying (i) a relatively high *S*_c/o_, similar to that of woody evergreen species from xeric habitats [14]; (ii) a higher affinity for CO_2_ (low *K*_c_), which ranks coffee Rubisco with the third lowest value of *K*_c_ among C_3_ and C_4_ plants recorded to date [69]; and (iii) a relatively high *k*_cat_^c^, superior to the average of species from warm climates [70]. We next modelled the responses of *A* as a function of *C*_c_ by comparing these Rubisco properties with those of several C_3_ species (Table S1) including *Limonium gibertii*, the species with the highest *S*_c/o_ and lowest *K*_c_ reported to date, thus particularly adapted to low *C*_c_ [14]. Importantly, we found that, all else being equal, the *A* values that would be achieved using the Rubisco kinetic properties of coffee or *Limonium* would be quite similar and superior to those from all other species analysed (Figure 2) suggesting that coffee presents an “efficient” Rubisco well-tuned for operating at low *C*_c_. Additionally, the higher *k*_cat_^c^ and lower *K*_c_ mean that fewer Rubisco molecules are required to realise a given *A*. Nevertheless, given that lower *K*_c_ leads to a reduction in *C*_c_trans_, *A* of coffee leaves is expected to begin to be limited by RuBP regeneration at relatively low *C*_c_, which could, to some extent, reduce the revenue stream in a scenario of increasing CO_2_ atmospheric levels.

The rates of respiration and photorespiration are other key processes influencing the plant carbon balance. In sun plants, *R*_D_ and *R*_P_ corresponded to 10% and 38% of *A*, respectively. However, given that *R*_L_ represented only a fraction (25%) of *R*_D_, which is in line with the inhibition of mitochondrial respiration in the presence of light [71], [72], the realised constraint of respiration on *A* is expected to be relatively low. In turn, *R*_P_ is expected to significantly affect *A* at midday, when the stomatal closure in coffee leaves exacerbates the drawdown from *C*_a_ to *C*_i_ [24], thus favouring the oxygenase activity of Rubisco.

## Conclusion

Regardless of the light treatments, *A* was mainly limited by stomatal factors followed by similar limitations associated with the mesophyll and biochemical constraints. Our data suggest that an increased *g*_m_/*g*_s_ ratio coupled with a Rubisco well-tuned for operating at low *C*_c_ might be adaptations to a lower *C*_i_ resulting from the low *g*_s_; these adaptations might have helped the establishment of coffee plants when they were moved from the understoreys to the more stressful conditions of open fields, where high vapour pressure deficit and elevated temperatures may constrain gas exchange. Our results also suggest that the coffee plant does not properly optimise its resource allocation: on the one hand, there seems to be an over-investment in capacity for carboxylation and electron transport despite limited light availability under shade; on the other hand, an excess of electron transport capacity is to be expected under full sun. Nevertheless, this excess might be useful in attempts to increase *A* in coffee through breeding aimed at improving hydraulic traits (*D*_v_ and *K*_L_) and expectedly supporting a high *g*_s_. Finally, we contend that the large diffusive resistance should lead to large drawdown from *C*_a_ to *C*_c_, thus favouring the occurrence of relatively high *R*_p_ despite the relatively high *S*_c/o_ of coffee Rubisco, which ultimately leads to further limitations to *A*.

## Supporting Information

Figure S1 **The relationship between sub-stomatal CO_2_ concentration and mesophyll-to-stomatal conductance ratio in the multi-species dataset from Flexas et al.** **[63].**
(TIF)

Figure S2 **The response of net photosynthetic rate and electron transport rate to the photosynthetic photon flux density in coffee plants developed under shade or full sun.**
(TIF)

Table S1 **The Rubisco kinetic constants for several C_3_ species.**
(DOCX)

## References

[1] Brodribb TJ, Holbrook NM, Zwieniecki MA, Palma B (2005) Leaf hydraulic capacity in ferns, conifers and angiosperms: impacts on photosynthetic maxima. New Phytol 165: 839–846. 15720695 10.1111/j.1469-8137.2004.01259.x

[2] Sack L, Cowan PD, Jaikumar N, Holbrook NM (2003) The ‘hydrology’ of leaves: co-ordination of structure and function in temperate woody species. Plant Cell Environ 26: 1343–1356.

[3] Sack L, Frole K (2006) Leaf structural diversity is related to hydraulic capacity in tropical rain forest trees. Ecology 87: 483–491. 16637372 10.1890/05-0710

[4] Brodribb TJ (2009) Xylem hydraulic physiology: The functional backbone of terrestrial plant productivity. Plant Sci 177: 245–251.

[5] Brodribb TJ, Feild TS, Jordan GJ (2007) Leaf maximum photosynthetic rate and venation are linked by hydraulics. Plant Physiol 144: 1890–1898. 17556506 10.1104/pp.107.101352 PMC1949879

[6] Carins Murphy MR, Jordan GJ, Brodribb TJ (2012) Differential leaf expansion can enable hydraulic acclimation to sun and shade. Plant Cell Environ 35: 1407–1418. 22339445 10.1111/j.1365-3040.2012.02498.x

[7] Warren CR (2008) Stand aside stomata, another actor deserves centre stage: the forgotten role of the internal conductance to CO_2_ transfer. J Exp Bot 59: 1475–1487. 17975206 10.1093/jxb/erm245

[8] Flexas J, Barbour MM, Brendel O, Cabrera HM, Carriquí M, et al (2012) Mesophyll diffusion conductance to CO_2_: an unappreciated central player in photosynthesis. Plant Sci 193–194: 70–84. 10.1016/j.plantsci.2012.05.009 22794920

[9] Ferrio JP, Pou A, Florez-Sarasa I, Gessler A, Kodama N, et al (2012) The Péclet effect on leaf water enrichment correlates with leaf hydraulic conductance and mesophyll conductance for CO_2_ . Plant Cell Environ 35: 611–625. 21988489 10.1111/j.1365-3040.2011.02440.x

[10] Farquhar GD, von Caemmerer S, Berry JA (1980) A biochemical model of photosynthetic CO_2_ assimilation in leaves of C3 species. Planta 149: 78–90. 24306196 10.1007/BF00386231

[11] Ethier GJ, Livingston NJ (2004) On the need to incorporate sensitivity to CO_2_ transfer conductance into the Farquhar-von Caemmerer-Berry leaf photosynthesis model. Plant Cell Environ 27: 137–153.

[12] Niinemets Ü, Díaz-Espejo A, Flexas J, Galmés J, Warren CR (2009) Role of mesophyll diffusion conductance in constraining potential photosynthetic productivity in the field. J Exp Bot 60: 2249–2270. 19395391 10.1093/jxb/erp036

[13] von Caemmerer S (2000) Biochemical models of leaf photosynthesis. CSIRO Publishing: Canberra.

[14] Galmés J, Flexas J, Keys AJ, Cifre J, Mitchell RAC, et al (2005) Rubisco specificity factor tends to be larger in plant species from drier habitats and in species with persistent leaves. Plant Cell Environ 28: 571–579.

[15] Galmés J, Conesa MAN, Ochogavía JM, Perdomo JA, Francis DM, et al (2011) Physiological and morphological adaptations in relation to water use efficiency in Mediterranean accessions of *Solanum lycopersicum* . Plant Cell Environ 34: 245–260. 20955222 10.1111/j.1365-3040.2010.02239.x

[16] Tcherkez GGB, Farquhar GD, Andrews TJ (2006) Despite slow catalysis and confused substrate specificity, all ribulose bisphosphate carboxylases may be nearly perfectly optimized. Proc Natl Acad Sci USA 103: 7246–7251. 16641091 10.1073/pnas.0600605103 PMC1464328

[17] Farquhar GD, von Caemmerer S (1982) Modeling of photosynthetic responses to environmental conditions. In: Lange OL, Nobel PS, Osmond CB, Ziegler H, editors. Encyclopedia of plant physiology (New Series). Springer-Verlag: Berlin, 549–587.

[18] DaMatta FM, Ronchi CP, Maestri M, Barros RS (2010) Coffee: environment and crop physiology. In: DaMatta FM, editor. Ecophysiology of tropical tree crops. Nova Science Publishers: New York, 181–216.

[19] DaMatta FM (2004) Ecophysiological constraints on the production of shaded and unshaded coffee: a review. Field Crops Res 86: 99–114.

[20] Franck N, Vaast P, Génard M, Dauzat J (2006) Soluble sugars mediate sink feedback down-regulation of leaf photosynthesis in field-grown *Coffea arabica.* . Tree Physiol 26: 517–525. 16414930 10.1093/treephys/26.4.517

[21] Silva EA, DaMatta FM, Ducatti C, Regazzi AJ, Barros RS (2004) Seasonal changes in vegetative growth and photosynthesis of arabica coffee trees. Field Crops Res 89: 349–357.

[22] Ceulemans R, Saugier B (1993) Photosynthesis. In: Raghavendra AS, editor. Physiology of trees. John Wiley: New York, 21–50.

[23] Araújo WL, Dias PC, Moraes GABK, Celin EF, Cunha RL, et al (2008) Limitations to photosynthesis in coffee leaves from different canopy positions. Plant Physiol Biochem 46: 884–890. 18603439 10.1016/j.plaphy.2008.05.005

[24] Batista D, Araújo WL, Antunes WC, Cavatte PC, Moraes GABK, et al (2012) Photosynthetic limitations in coffee plants are chiefly governed by diffusive factors. Trees 26: 459–468.

[25] Galmés J, Medrano H, Flexas J (2007) Photosynthetic limitations in response to water stress and recovery in Mediterranean plants with different growth forms. New Phytol 175: 81–93. 17547669 10.1111/j.1469-8137.2007.02087.x

[26] DaMatta FM (2010) Introduction. In: DaMatta FM, editor. Ecophysiology of tropical tree crops. Nova Science Publishers: New York, 1–6.

[27] Martins SCV, Galmés JG, Molins A, DaMatta FM (2013) Improving the estimation of mesophyll conductance: on the role of electron transport rate correction and respiration. J Exp Bot 64: 3285–3298. 23833194 10.1093/jxb/ert168 PMC3733151

[28] DaMatta FM, Maestri M, Barros RS (1997) Photosynthetic performance of two coffee species under drought. Photosynthetica 34: 257–264.

[29] Salisbury EJ (1927) On the causes and ecological significance of stomatal frequency with special reference to the woodland flora. Philos Trans R Soc Lond B Biol Sci 216: 1–65.

[30] Scoffoni C, Rawls M, McKown A, Cochard H, Sack L (2011) Decline of leaf hydraulic conductance with dehydration: relationship to leaf size and venation architecture. Plant Physiol 156: 832–843. 21511989 10.1104/pp.111.173856 PMC3177279

[31] Antunes WC, Pompelli MF, Carretero DM, DaMatta FM (2008) Allometric models for non-destructive leaf area estimation in coffee (*Coffea arabica* and *C. canephora*). Ann Appl Biol 153: 33–40.

[32] Lewis AM, Boose ER (1995) Estimating volume flow rates through xylem conduits. Am J Bot 82: 1112–1116.

[33] Franks PJ, Drake PL, Beerling DJ (2009) Plasticity in maximum stomatal conductance constrained by negative correlation between stomatal size and density: an analysis using *Eucalyptus globules* . Plant Cell Environ 32: 1737–1748. 19682293 10.1111/j.1365-3040.2009.02031.x

[34] Franks P, Farquhar G (2001) The effect of exogenous abscisic acid on stomatal development, stomatal mechanics, and leaf gas exchange in *Tradescantia virginiana* . Plant Physiol 125: 935–942. 11161050 10.1104/pp.125.2.935 PMC64894

[35] Brodribb TJ, Holbrook MN (2003) Stomatal closure during leaf dehydration, correlation with other leaf physiological traits. Plant Physiol 132: 2166–2173. 12913171 10.1104/pp.103.023879 PMC181300

[36] Cavatte PC, Oliveira AAG, Morais LE, Martins SCV, Sanglard LMVP, et al (2012) Could shading reduce the negative impacts of drought on coffee? A morphophysiological analysis. Physiol Plant144: 111–122. 10.1111/j.1399-3054.2011.01525.x 21939445

[37] Genty B, Briantais JM, Baker NR (1989) The relationship between the quantum yield of photosynthetic electron-transport and quenching of chlorophyll fluorescence. Biochim Biophys Acta 990: 87–92.

[38] Valentini R, Epron D, Angelis P, Matteucci G, Dreyer E (1995) In situ estimation of net CO_2_ assimilation, photosynthetic electron flow and photorespiration in Turkey oak (*Q. cerris* L.) leaves: diurnal cycles under different levels of water supply. Plant Cell Environ 18: 631–640.

[39] Laisk AK (1977) Kinetics of photosynthesis and photorespiration in C3 plants. Nauka: Moscow.

[40] Harley PC, Loreto F, Di Marco G, Sharkey TD (1992) Theoretical considerations when estimating the mesophyll conductance to CO_2_ flux by analysis of the response of photosynthesis to CO_2_ . Plant Physiol 98: 1429–1436. 16668811 10.1104/pp.98.4.1429 PMC1080368

[41] Rodeghiero M, Niinemets Ü, Cescatti A (2007) Major diffusion leaks of clamp-on leaf cuvettes still unaccounted: how erroneous are the estimates of Farquhar et al. model parameters? Plant Cell Environ 30: 1006–1022. 17617828 10.1111/j.1365-3040.2007.001689.x

[42] Gu L, Pallardy SG, Tu K, Law BE, Wullschleger SD (2010) Reliable estimation of biochemical parameters from C_3_ leaf photosynthesis-intercellular carbon dioxide response curves. Plant Cell Environ 33: 1852–1874. 20561254 10.1111/j.1365-3040.2010.02192.x

[43] Grassi G, Magnani F (2005) Stomatal, mesophyll conductance and biochemical limitations to photosynthesis as affected by drought and leaf ontogeny in ash and oak trees. Plant Cell Environ 28: 834–849.

[44] Sack L, Holbrook MN (2006) Leaf hydraulics. Annu Rev Plant Biol 57: 361–381. 16669766 10.1146/annurev.arplant.56.032604.144141

[45] Casson SA, Franklin KA, Gray JE, Grierson CS, Whitelam GC, et al (2009) Phytochrome B and PIF4 regulate stomatal development in response to light quantity. Curr Biol 19: 229–234. 19185498 10.1016/j.cub.2008.12.046

[46] Chaturvedi RK, Raghubanshi AS, Singh JS (2011) Leaf attributes and tree growth in a tropical dry forest. J Veg Sci 22: 917–931.

[47] Nogueira A, Martinez CA, Ferreira LL, Prado CHBA (2004) Photosynthesis and water use efficiency in twenty tropical tree species of differing succession status in a Brazilian reforestation. Photosynthetica 42: 351–356.

[48] Gascó A, Nardini A, Salleo S (2004) Resistance to water flow through leaves of *Coffea arabica* is dominated by extra-vascular tissues. Funct Plant Biol 31: 1161–1168. 10.1071/FP04032 32688983

[49] Sack L, Tyree MT, Holbrook NM (2005) Leaf hydraulic architecture correlates with regeneration irradiance in tropical rainforest trees. New Phytol 167: 403–413. 15998394 10.1111/j.1469-8137.2005.01432.x

[50] Boyce CK, Lee J-E, Field TS, Brodribb TJ, Zwieniecki MA (2010) Angiosperms helped put the rain in the rainforests: The impact of plant physiological evolution on tropical biodiversity. Ann Missouri Bot Gard 97: 527–540.

[51] Brodribb TJ, Feild TS, Sack L (2010) Viewing leaf structure and evolution from a hydraulic perspective. Funct Plant Biol 37: 488–498.

[52] Galmés J, Ochogavía JM, Gago J, Roldán EJ, Cifre J, et al (2013) Leaf responses to drought stress in Mediterranean accessions of *Solanum lycopersicum*: anatomical adaptations in relation to gas exchange parameters. Plant Cell Environ 36: 920–935. 23057729 10.1111/pce.12022

[53] Russo SE, Cannon WL, ElowskyC, Tan S, Davies SJ (2010) Variation in leaf stomatal traits of 28 tree species in relation to gas exchange along an edaphic gradient in a Bornean rain forest. Am J Bot 97: 1109–1120. 21616863 10.3732/ajb.0900344

[54] Eensalu E, Kupper P, Sellin A, Rahi M, Sõber A, et al (2010) Do stomata operate at the same relative opening range along a canopy profile of *Betula pendula* ? Funct Plant Biol 35: 103–110. 10.1071/FP07258 32688761

[55] Carins Murphy MR, Jordan GJ, Brodribb TJ (2013) Acclimation to humidity modifies the link between leaf size and the density of veins and stomata. Plant Cell Environ 37: 124–131. 23682831 10.1111/pce.12136

[56] Hanba Y, Kogami H, Terashima I (2003) The effect of internal CO_2_ conductance on leaf carbon isotope ratio. Isotopes Environ Health Studies 39: 5–13. 10.1080/1025601031000102233 12812251

[57] Manter DK, Kerrigan J (2004) *A*/*C* _i_ curve analysis across a range of woody plant species: influence of regression analysis parameters and mesophyll conductance. J Exp Bot 55: 2581–2588. 15501912 10.1093/jxb/erh260

[58] Warren CR, Adams MA (2006) Internal conductance does not scale with photosynthetic capacity: implications for carbon isotope discrimination and the economics of water and nitrogen use in photosynthesis. Plant Cell Environ 29: 192–201. 17080635 10.1111/j.1365-3040.2005.01412.x

[59] Flexas J, Ribas-Carbó M, Díaz-Espejo A, Galmés J, Medrano H (2008) Mesophyll conductance to CO_2_: current knowledge and future prospects. Plant Cell Environ 31: 602–621. 17996013 10.1111/j.1365-3040.2007.01757.x

[60] Terashima I, Hanba YT, Tholen D, Niinemets Ü (2011) Leaf functional anatomy in relation to photosynthesis. Plant Physiol 155: 108–116. 21075960 10.1104/pp.110.165472 PMC3075775

[61] Monti A, Bezzi G, Venturi G (2009) Internal conductance under different light conditions along the plant profile of Ethiopian mustard (*Brassica carinata* A. Brown.). J Exp Bot 60: 2341–2350. 19237547 10.1093/jxb/erp032

[62] Warren CR, Löw M, Matyssek R, Tausz M (2007) Internal conductance to CO_2_ transfer of adult *Fagus sylvatica*: Variation between sun and shade leaves and due to free-air ozone fumigation. Environ Exp Bot 59: 130–138.

[63] Flexas J, Niinemets Ü, Gallé A, Baubour MM, Centritto M, et al (2013) Diffusional conductances to CO_2_ as a target for increasing photosynthesis and photosynthetic water-use efficiency. Photosynth Res 117: 45–59. 23670217 10.1007/s11120-013-9844-z

[64] Ronquim JC, Prado CHBA, Novaes P, Fahl JI, Ronquim CC (2006) Carbon gain in *Coffea arabica* during clear and cloudy days in the wet season. Exp Agric 42: 147–164.

[65] Chaves ARM, Ten-Caten A, Pinheiro HA, Ribeiro A, DaMatta FM (2008) Seasonal changes in photoprotective mechanisms of leaves from shaded and unshaded field-grown coffee (*Coffea arabica* L.). Trees 22: 351–361.

[66] Chaves ARM, Martins SCV, Batista KD, Celin EF, DaMatta FM (2012) Varying leaf-to-fruit ratios affect branch growth and dieback, with little to no effect on photosynthesis, carbohydrate or mineral pools, in different canopy positions of field-grown coffee trees. Environ Exp Bot 77: 207–218.

[67] DaMatta FM, Cunha RL, Antunes WC, Martins SVC, Araújo WL, et al (2008) In field-grown coffee trees source-sink manipulation alters photosynthetic rates, independently of carbon metabolism, via alterations in stomatal function. New Phytol 178: 348–357. 18266616 10.1111/j.1469-8137.2008.02367.x

[68] Niinemets Ü, Kull O, Tenhunen JD (1998) An analysis of light effects on foliar morphology, physiology, and light interception in temperate deciduous woody species of contrasting shade tolerance. Tree Physiol 18: 681–696. 12651418 10.1093/treephys/18.10.681

[69] Savir Y, Noor E, Milo R, Tlusty T (2010) Cross-species analysis traces adaptation of Rubisco toward optimality in a low-dimensional landscape. Proc Natl Acad Sci USA 107: 3475–3480. 20142476 10.1073/pnas.0911663107 PMC2840432

[70] Sage RF, Cen Y, Li M (2002) The activation state of Rubisco directly limits photosynthesis at low CO_2_ and low O_2_ partial pressures. Photosynth Res 71: 241–250. 16228135 10.1023/A:1015510005536

[71] Tcherkez G, Cornic G, Bligny R, Gout E, Ghashghaie J (2005) *In vivo* respiratory metabolism of illuminated leaves. Plant Physiol 138: 1596–1606. 15980193 10.1104/pp.105.062141 PMC1176429

[72] Tcherkez G, Bligny R, Gout E, Mahé A, Hodges M, et al (2008) Respiratory metabolism of illuminated leaves depends on CO_2_ and O_2_ conditions. Proc Natl Acad Sci USA 105: 797–802. 18184808 10.1073/pnas.0708947105 PMC2206616

