# Supplementary figures and images for: Understanding the Low Photosynthetic Rates of Sun and Shade Coffee Leaves: Bridging the Gap on the Relative Roles of Hydraulic, Diffusive and Biochemical Constraints to Photosynthesis
Source: PLoS One. 2014 Apr 17;9(4):e95571. doi: 10.1371/journal.pone.0095571 (PMC3990704; doi:10.1371/journal.pone.0095571)

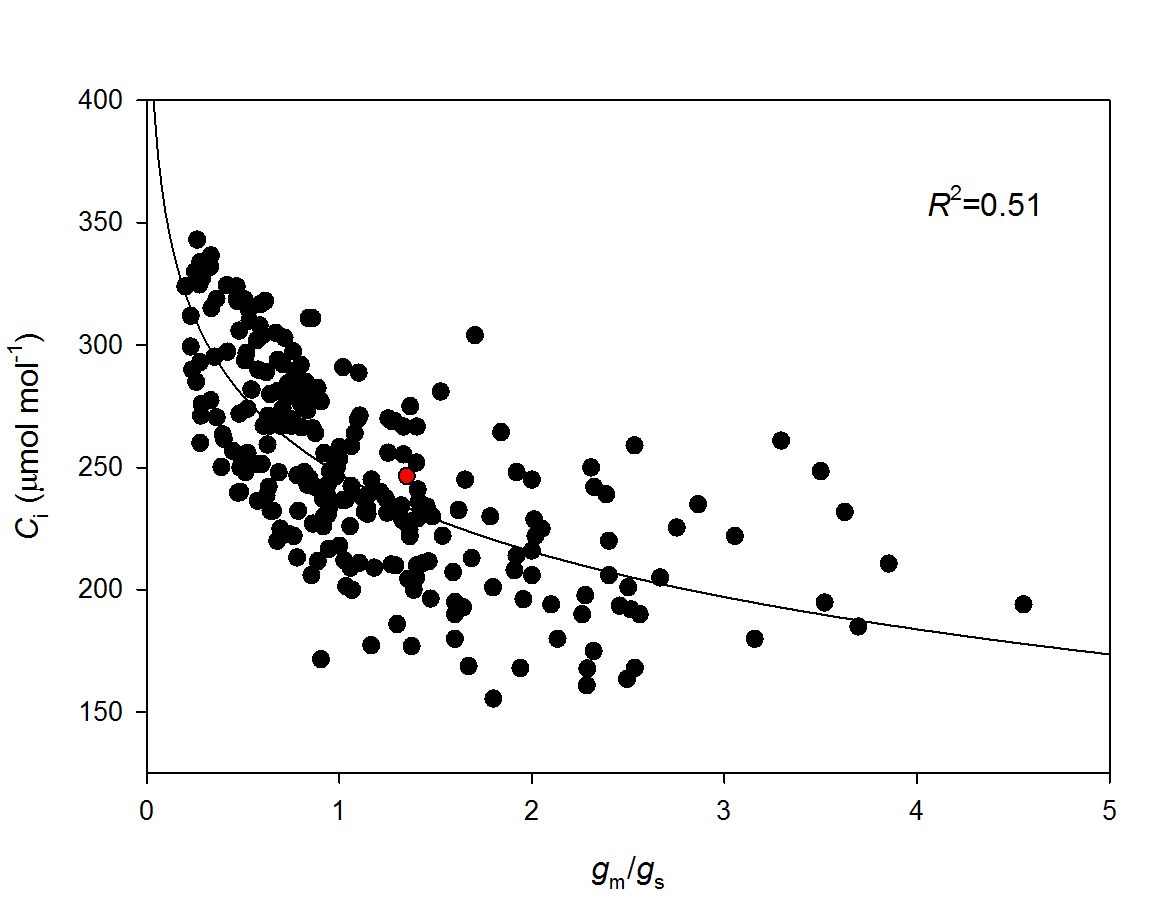

Supplement: Figure S1 — The relationship between sub-stomatal CO2 concentration and mesophyll-to-stomatal conductance ratio in the multi-species dataset from Flexas et al. [63]. (TIF) [file pone.0095571.s001.tif]
