# Supplementary material for: Understanding the Low Photosynthetic Rates of Sun and Shade Coffee Leaves: Bridging the Gap on the Relative Roles of Hydraulic, Diffusive and Biochemical Constraints to Photosynthesis
Source: PLoS One. 2014 Apr 17;9(4):e95571. doi: 10.1371/journal.pone.0095571 (PMC3990704; doi:10.1371/journal.pone.0095571)
Supplement: Table S1 — The Rubisco kinetic constants for several C3 species. (DOCX) [file pone.0095571.s003.docx]

**Table S1.**  Rubisco kinetic constants for the other C_3_ species^a^.

| Species | *S*_c/o_ | Γ* (μbar) | *K*_c_ (μM) | *K*_o_ (μM) | *k*_cat_^c^(s^-1^) |
| --- | --- | --- | --- | --- | --- |
| *Limonium gibertii* | 110.5 ± 1.6 | 35.2 ± 0.5 | 8.9 ± 0.5 | 593 ± 75 | 2.7 ± 0.8 |
| *Chenopodium album* | 78.7 ± 1.0 | 49.6 ± 0.6 | 11.2 ± 2.8 | 415 ± 16 | 2.9 ± 0.1 |
| *Spinacia oleracea* | 79.8. ± 0.5 | 48.9 ± 0.3 | 12.1 ± 0.8 | 574 ± 19 | 3.2 ± 0.1 |
| *Triticum aestivum* | 90.0 ± 1.0 | 43.3 ± 0.5 | 14.0 ± 3.0 | 730 ± 41 | 2.5 ± 0.2 |

Abbreviations: *S*_c/o_, Rubisco specificity factor; Γ*, CO_2_ compensation point in the absence of mitochondrial respiration; *K*_c_ and *K*_o_, the Michaelis-Menten kinetics for CO_2_ and O_2_, respectively; *k*_cat_^c^, Rubisco catalytic turnover rate for the carboxylase reaction. ^a^ *Nicotiana tabacum* and *Flaveria pringlei* (two additional C_3_ species from the Savir et al. [69] dataset) were excluded from the analysis because their curves were nearly identical to the other curves already presented in Figure 2.
